# Adaptation to Complex Pictures: Exposure to Emotional Valence Induces Assimilative Aftereffects

**DOI:** 10.3389/fpsyg.2017.00054

**Published:** 2017-01-30

**Authors:** Rocco Palumbo, Stefania D’Ascenzo, Angelica Quercia, Luca Tommasi

**Affiliations:** ^1^Schepens Eye Research Institute, Harvard Medical School, BostonMA, USA; ^2^Department of Psychological Science, Humanities and Territory, “G. d’Annunzio” UniversityChieti, Italy; ^3^Department of Philosophy and Communication, University of BolognaBologna, Italy; ^4^Department of Neuroscience, Imaging and Clinical Sciences, “G. d’Annunzio” UniversityChieti, Italy

**Keywords:** adaptation, aftereffect, positive valence, negative valence, complex images

## Abstract

Aftereffects have been documented for a variety of perceptual categories spanning from body gender to facial emotion, thus becoming an important tool in the study of high-level vision and its neural bases. We examined whether the perceived valence of a complex scene is subject to aftereffects, by observing the participants’ evaluation of the valence of a test picture preceded by a different picture. For this study, we employed an adaptation paradigm with positive and negative images used as adapters, and positive, negative, and neutral images used as tests. Our results show that adaptation to complex emotional pictures induces assimilative aftereffects: participants judged neutral tests more positively following positive adapters and more negatively following negative adapters. This depended on the prolonged adaptation phase (10 s), as the results of a second experiment, in which adapters lasted for 500 ms, did not show aftereffects. In addition, the results show that assimilative aftereffects of negative and positive adapters also manifested themselves on non-neutral (negative and positive) targets, providing evidence that the global emotional content of complex pictures is suitable to induce assimilative aftereffects.

## Introduction

Aftereffects produced by the prolonged exposure to certain stimuli are a well-known phenomenon in the perceptual literature. Since Aristotle, it has been observed that maintaining fixation for a long time (adaptation) on a specific stimulus’ property produces a perceptual change (aftereffect) of that specific property in a following stimulus. These effects can be subdivided into assimilative aftereffects or contrastive aftereffects (Howard and Rogers, 1995). The former type is a perceptual change on the second stimulus (named here the test) that runs in the same direction of the first stimulus (named here the adapter). The later type is a perceptual change on a test stimulus that runs in the opposite direction of the adapter one. One relevant factor to determine whether assimilation or contrastive effects prevail is stimulus type. Strong, salient, high-contrast, long-duration stimuli tend to lead to contrast effects, while brief, less salient low-contrast stimuli lead to assimilative effects (Pantle et al., 2000; Kanai and Verstraten, 2005; Yoshimoto and Takeuchi, 2013). In particular, contrastive aftereffects are ubiquitous in both low- and high-level perception: the well-known “waterfall illusion,” an example of a motion aftereffect, is just an example. The biological explanation of this phenomenon assumes that prolonged exposure to an object’s perceptual feature (such as its motion direction) reduces the responses of the neurons tuned to that particular feature, facilitating the perception of the feature in the opposite direction along its continuum (e.g., Barlow and Hill, 1963; Srinivasan and Dvorak, 1979; Huk et al., 2001; Thompson and Burr, 2009; Glasser et al., 2011). Many simple properties have been investigated besides motion direction, such as color (e.g., McCollough, 1965), orientation (e.g., Gibson and Radner, 1937; Dekel and Sagi, 2015), size (e.g., Blakemore and Campbell, 1969; Blakemore and Sutton, 1969), contrast, and shape (e.g., Suzuki and Cavanagh, 1998).

Aftereffects, together with other visual illusions and phenomena, represent a powerful instrument in understanding the processes that intervene among sensation, neural coding of the environment, and perception. For this reason, in recent decades, several research studies have extended the investigation to observe whether visual aftereffects occur for more complex properties, not strictly related to the physical features of the stimulus, but for more high-level cognitive representations, in particular for socially relevant properties of faces and bodies, such as gender (e.g., Webster et al., 2004; Palumbo et al., 2013; D’Ascenzo et al., 2014), age (e.g., Schweinberger et al., 2010), body representation (Cazzato et al., 2016), and emotional expression (e.g., Fox and Barton, 2007). For example, after prolonged exposure to male faces, neutral faces that were subsequently presented appeared female to the observer and vice versa (Webster et al., 2004). To further support the evidence that aftereffects can be unrelated to the physical features of the stimulus to which the subject is adapted, numerous studies have tried to investigate whether aftereffects occur across categories as well (e.g., Davidenko et al., 2008; Ghuman et al., 2010; Javadi and Wee, 2012; Kessler et al., 2013). In a study by Palumbo et al. (2015), for instance, the authors found that 10 s of exposure to male or female faces produces a gender aftereffect on the following presentation of androgynous bodies. This example suggests that adaptation to gender is not related to the physical features of the stimulus, but to the representation of gender as a more global property of bodies and faces. It should be noted that Kovács et al. (2006) investigated gender aftereffects using faces and body parts as stimuli, finding that prolonged adaptation to hands or faces produces aftereffects on hand or face test stimuli, respectively. However, no aftereffects were found when the adaptor and test stimuli belonged to different categories, suggesting that the underlying adaptation mechanisms are category-specific. Although several studies on aftereffects of socially relevant stimuli have strengthened the evidence that these effects extend to complex perceptual contents, no study has investigated the role of adaptation to more complex content on subsequent perceptions. The perceived emotional content of pictures appears as a potential candidate in the study of this phenomenon, one in which would possibly allow to carefully exclude the well-known contribution of expressive faces, that may drive the presence of aftereffects.

The ability to perceive and classify stimuli as positive or negative is one of the most crucial abilities for survival (Damasio, 1994). In order to quickly detect hazards and respond properly to the environment, it is important to make sense of the surroundings by assigning emotional value to the sensory input (Greene et al., 2015; Zebrowitz et al., 2015; Altamura et al., 2016; Mammarella et al., 2016a). That means, we relentlessly create an internal mental representation of the stimuli immediately present in our surroundings by categorizing the scene as positive or negative in valence (for a review, see Brosch et al., 2010; Mammarella et al., 2016b). Regarding the temporal dimension, which is a crucial factor in assessing any perceptual content, the effects due to the duration of exposure and the order of presentation when multiple items are presented, are aspects that have been investigated very thoroughly. However, they have been investigated in rather compartmentalized traditions belonging to perception, cognition, learning, and in applied domains, such as clinical psychology or even marketing studies. Picture valence is no exception: the effects of previous exposure to emotional stimuli have been widely investigated through several experimental paradigms, such as priming (e.g., Meyer and Schvaneveldt, 1971; Neely, 1991; Klauer and Musch, 2003), habituation (e.g., Leventhal et al., 2007), and mood conditioning (e.g., Sutton et al., 1997; Di Domenico et al., 2015, 2016). Furthermore, exposure therapy (Abramowitz, 2013), can be listed as one of the potential clinical applications of these phenomena.

However, there is a scarcity of studies that have investigated whether and how the perceived valence of complex images might be affected by adaptation to previous images that have themselves a positive or a negative valence. These types of adaptation studies would provide evidence that the emotional impression (valence) arising from any window on the environment may represent a high-level category in perceptual analysis, beyond the well-known evidence concerning the specific aspect of facial emotion adaptation. The fact that adaptation to primary and secondary features, such as orientation or motion of simple stimuli, can produce aftereffects or, that adaptation to higher features, such as facial expressions, similarly produces aftereffects, can have a twofold relevance. Firstly, it could help in defining which features, or combinations thereof, can qualify as genuine perceptual categories (if adaptation to body gender is observed, then gender should have a high likelihood to be deemed in itself a perceptual category). Secondly, if neural mechanisms are invoked to explain the existence of adaptation phenomena across a wide hierarchy of content complexity (from low-level to high-level), then one can conclude that specific neural units or regions must be responsible for encoding those types of content, providing indirect evidence of content-specific brain specialization. Although not all would necessarily endorse the second argument, we believe that measuring adaptation of the perceived valence arising from pictures could be a test-bench for exploring the upper limits of perceptual adaptation, thus providing inputs for better understanding the notion of adaptation itself, its generality, and possibly its neural realization.

For this reason, in the present study we aimed to investigate whether adaptation to complex scenes with emotional content would affect the perception of subsequent scenes. We hypothesized that the attribution of valence to a complex scene, due to its relevance in eliciting quick behavioral responses crucial for survival (Damasio, 1994), may qualify as a genuine perceptual category. It has to be highlighted that valence is a continuous dimension, ranging from extremely negative to extremely positive. However, the valence of a complex pictorial input might be synthetically attributed by assigning one single value along that continuum. With these premises in mind, we conjectured that testing the susceptibility of valence attribution to adaptation, could act as a proxy of testing whether valence is a perceptual category. Furthermore, if the perceived valence is subject to aftereffect it would then be possible to hypothesize that the perceiver creates a representation of the valence contained in the scene. In order to test our hypothesis, we used an adaptation paradigm where we asked participants to look for 10 s (adaptation phase) at a complex scene, which had either a positive or negative emotional valence, and to rate the valence of a following scene (which could be positive, neutral, or negative). In current literature on adaptation and aftereffects, it is still unclear whether aftereffects can be considered due to a decisional bias. Based on this approach, the adapter itself is responsible for modulating the participant’s strategies of categorization, thus generating a sort of strategy rule where the subject tends to judge the test as opposite to the adapter just because it is different from it (Storrs, 2015). In order to exclude a possible decisional bias, we addressed this issue by asking our participants to rate the images, after the adaptation phase, rather than categorize them. Using this method, we thought that any change in the participant’s judgment would be attributed to the effect of the adaptation and not to a change of strategy in the categorization process. Moreover, because valence is a continuous dimension that can be quickly processed by individuals, we based our study on the fact that it could be used as a dimension onto which aftereffects could be precisely assessed as contrastive or assimilative, using a typical adaptation paradigm. Although a typical adaptation paradigm would use test stimuli that are neutral on a chosen continuum (e.g., an androgynous face in a male-female space), we used positive, negative, and neutral test stimuli (see also Fujimura et al., 2013; Czekóová et al., 2015). Differently from previous studies on consecutive effects on affective evaluation of pictures (Flaisch et al., 2008; Schupp et al., 2012; Fujimura et al., 2013; Czekóová et al., 2015), the pictures chosen as adapters and tests were deliberately selected to not include images of faces. This was crucial in order to avoid the possibility that any effect possibly observed could be driven by this specific category.

## Experiment 1

### Methods

#### Participants

Forty-nine volunteers (26 females; mean age: 20.42; SD: 3.11) were recruited at the University of Chieti (Italy) to take part in the experiment. All of the participants had normal or corrected-to-normal vision. The local ethics committee approved this study and written informed consent was obtained from all participants. All procedures employed in the study comply with ethical standards on human experimentation and with the Helsinki Declaration of 1975, as revised in 2008.

#### Stimuli

All stimuli were images taken from the International Affective Picture System (IAPS; Lang et al., 2008) with a positive, negative, or neutral valence. Furthermore, it should be noted that images containing faces were explicitly excluded to avoid the well-known effect of these stimuli on adaptation (e.g., Leopold et al., 2001).

Twenty-six independent raters (13 females; mean age: 22.15, range: 18–32) evaluated the valence of 176 images by means of a visual analog scale (VAS): the ends of the scale represented maximum negative value (-100%) on one side and maximum positive value (+100%) on the opposite side. The centre of the VAS represented neutrality (0). Following this rating, we selected 28 images: 11 positive, 11 negative, and 6 neutral; namely, the images that had received extreme mean scores in terms of positive and negative valence and the centremost mean scores in terms of neutral valence (the range of values of the positive, negative, and neutral images selected were +60% to +100%, -60 to -100%, and -4 to 4%, respectively). Among these selected images, five were used as positive adapters, five as negative adapters, and the remaining images were used as tests (six neutrals, *M* = -0.81%; six positive, *M* = +66.41%; six negative, *M* = -80.94%).

#### Procedure

**FIGURE 1:**
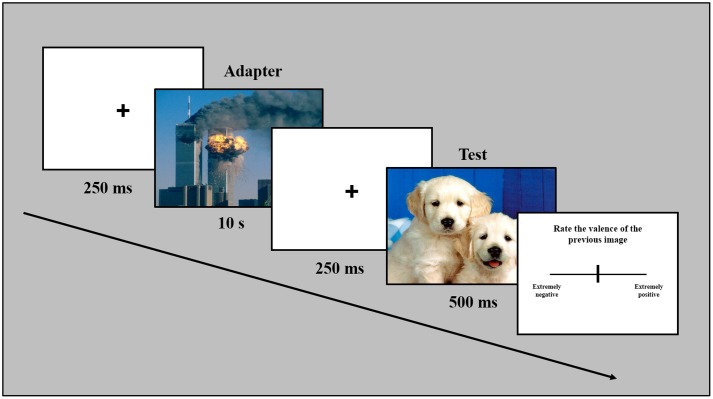
**Temporal sequence of a representative trial.** Note that stimuli are not drawn to scale. Sample pictures in this figure are taken from a different source to avoid showing International Affective Picture System (IAPS) pictures.

Participants were seated in a dark experimental room and images were presented on a 17-inch Philips 170S monitor via an E-prime 1.2-controlled script. In a representative trial of the experiment (**Figure 1**), a fixation cross was presented in the center of the screen for 250 ms. The fixation cross was followed by the presentation of the adapter full screen for 10 s. The adapter was followed by the presentation of a fixation cross (duration 250 ms). Subsequently, the test image was presented for 500 ms. After the test image disappeared, the participant evaluated the valence of the image just presented by providing the response on a VAS, i.e., a line (length 31°, thickness 0.2°) presented horizontally in the center of the screen, by moving a slider on the line with a mouse. The line represented a double-ended continuum where the two ends, labeled as “extreme negative” and “extreme positive,” indicated the maximum negative value (-100%) on one side and the maximum positive (+100%) value on the opposite side. For each trial, the slider was positioned in the center of the line (neutral value; 0%) in order to not influence the participant’s decision. The positive/negative or negative/positive spatial polarity of the continuum was balanced across participants. Each of the ten adapters (positive or negative) were presented in a random order and followed by a one of the 18 different tests stimuli (positive, negative, or neutral), for a total of 180 trials. Each new trial started after the participant’s last response. The experiment lasted approximately 60 min overall.

### Statistical Analyses

Scores were coded on a continuum, ranging from -100 (negative) to +100 (positive), and converted to percentage values. They were submitted to a mixed factorial Analysis of Variance (ANOVA) with Valence of the Adapter (levels: negative; positive) and Test (levels: negative; neutral; positive) as within-participant factors and Gender as between-participant factor. A *post hoc* analysis using the Duncan test was used for multiple comparisons. The level of statistical significance was set at 5% (*p* < 0.05).

In addition, single sample *t*-tests were computed separately for each test type (according to adaptation to positive and negative stimuli), in order to compare the judgment to the mean value obtained during the preliminary rating for each valence level. This analysis was carried out to assess whether the scores obtained following adaptation depended upon the presence of the adapter.

### Results

The analysis showed a significant main effect of Valence of the Adapter, *F*_(1,48)_ = 5.51, *MSE* = 2363, *p* < 0.05, $\eta_{p}^{2}$ = 0.11, with a more negative value after adaptation to a negative image (*M* = -5.84%; *SD* = 54.76) with respect to adaptation to a positive image (*M* = -0.06%; *SD* = 55.01). The main effect of Test was also significant, *F*_(1,48)_ = 447.27, *MSE* = 375113, *p* < 0.001, $\eta_{p}^{2}$ = 0.90, showing that irrespective of the Valence of the Adapter, participants gave a negative score to the negative test images (*M* = -67.09%; *SD* = 18.14), which significantly differed from that of the neutral test images (*M* = 1.20%; *SD* = 17.91; *p* < 0.001) and from that of the positive test images (*M* = 57.03; *SD* = 25.22; *p* < 0.001). Additionally, the score of the positive test images differed significantly from that of the neutral test images (*p* < 0.001).

**FIGURE 2:**
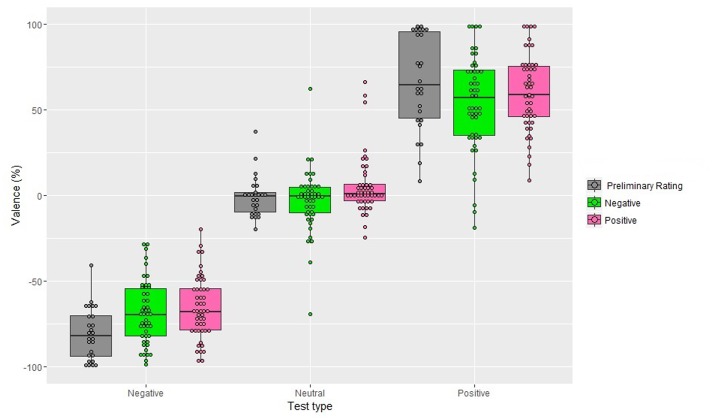
**Valence scores obtained in the preliminary rating and after 10 s of exposure to the adapter (Experiment 1), as a function of Valence of the Adapter and Test type.** Positive percentages values represent perceived valence and negative percentages represent perceived negative valence. The box plots show: the lower and upper quartile (the 25th and 75th percentiles, respectively); the whiskers, that refer to the minimum and the maximum values (excluding outliers) and the bold line, that refer to median value. Dots above the box plots are the mean rating of each participant. Dots outside whiskers indicate outliers.

The interaction between Valence of the Adapter and Test was significant, *F*_(1,48)_ = 3.61, *MSE* = 167.71, *p* < 0.05, $\eta_{p}^{2}$ = 0.07 (**Figure 2**). *Post hoc* comparisons showed that, considering the neutral test, the positive adapters shifted the judgment toward a more positive judgment (*M* = 5.38%; *SD* = 17.15) with respect to the negative adapters that shifted the judgment toward a more negative judgment (*M* = -2.98%; *SD* = 17.86; *p* < 0.001). The negative test was evaluated as more negative following the negative adapter (*M* = -68.60%; *SD* = 18.29) compared to the positive adapter (*M* = -65.58%; *SD* = 18.05; *p* < 0.05). Finally, the positive test was given a more positive judgment following the positive adapter (*M* = 60.01%; *SD* = 12.14) compared to when it was followed by the negative adapter (*M* = 54.03%; *SD* = 27.88; *p* < 0.001). Neither the main effect of Gender, *F*_(1,48)_ = 1.4, *p* = 0.25, nor the interactions involving this factor reached significance Valence of the Adapter and Gender: *F*_(1,48)_ = 0.5, *p* = 0.50; Test and Gender: *F*_(1,48)_ = 1, *p* = 0.36, Valence of the Adapter, Test and Gender: *F*_(1,48)_ = 1.2, *p* = 0.32.

In addition, single sample *t*-tests showed that the judgment given to the neutral tests after being adapted to the positive adapters was significantly more positive from the mean score of the neutral tests as obtained in the preliminary rating, *t*_(48)_ = 2.53, *p* < 0.05. The judgment given to the positive tests after being adapted to the positive and to the negative adapters was significantly less positive from the score given to the positive tests as obtained in the preliminary rating, *t*_(48)_ = -2.02, *p* < 0.05 and *t*_(48)_ = -3.10, *p* < 0.01, respectively. Moreover, the judgment given to the negative tests after being adapted to the positive adapters and after being adapted to the negative adapters were significantly less negative from the score given to the negative tests as obtained in the preliminary rating, *t*_(48)_ = 5.95, *p* < 0.001 and *t*_(48)_ = 4.72, *p* < 0.001, respectively. Finally, the judgment given to the neutral tests after being adapted to the negative adapters failed to reach significance when it was compared to the neutral tests’ score as obtained in the preliminary rating, *t*_(48)_ = -0.85, *p* = 0.39.

## Experiment 2

In order to ensure that the findings of Experiment 1 were the result of the prolonged exposure to adapter stimuli, we performed a second (control) experiment reducing the duration of the adapter to 500 ms. In this experiment, both the adapter and test had the same duration. However, the temporal duration of the adapter was too short to qualify the paradigm as an adaptation paradigm, but too long to qualify it as a priming paradigm.

### Methods

#### Participants

Twenty-seven new volunteers (15 females; mean age: 20.74; SD: 2.52) were recruited at the University of Chieti (Italy) to take part in the experiment. All of the participants had normal or corrected-to-normal vision. The local ethics committee approved this study and written informed consent was obtained from all participants. All procedures employed in the study comply with ethical standards on human experimentation and with the Helsinki Declaration of 1975, as revised in 2008.

#### Procedure

Stimuli, procedure, and statistical analysis were the same of that in Experiment 1, with the only exception that the adapter was presented for 500 ms.

### Results

The analysis showed a significant main effect of Test, *F*_(1,26)_ = 536.71, *MSE* = 25479, *p* < 0.001, $\eta_{p}^{2}$ = 0.95: irrespective of the Valence of the Adapter, participants gave a negative score to the negative test images (*M* = -79.81%; *SD* = 3.49), which significantly differed from that of the neutral test images (*M* = -0.69%; *SD* = 1.94; *p* < 0.001) and from that of the positive test images (*M* = 65.52%; *SD* = 2.39; *p* < 0.001). Additionally, the score of the positive test images differed significantly from that of the neutral test images (*p* < 0.001).

**FIGURE 3:**
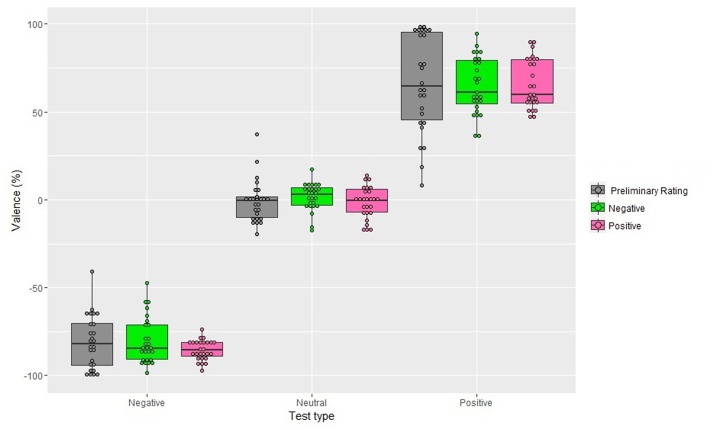
**Valence scores obtained in the preliminary rating and after 10 s of exposure to the adapter (Experiment 2), as a function of Valence of the Adapter and Test type.** Positive percentages values represent perceived valence and negative percentages represent perceived negative valence. The box plots show: the lower and upper quartile (the 25th and 75th percentiles, respectively); the whiskers, that refer to the minimum and the maximum values (excluding outliers) and the bold line, that refer to median value. Dots above the box plots are the mean rating of each participant. Dots outside whiskers indicate outliers.

The main effect of the Valence of the Adapter and its interaction with Test were not significant, *F*_(1,26)_ = 0, *p* = 0.99, *F*_(1,26)_ = 0.1, *p* = 0.92, respectively. Neither, the main effect of Gender, *F*_(1,26)_ = 0, *p* = 0.92, nor the interactions involving this factor reached significance, Valence of the Adapter and Gender: *F*_(1,26)_ = 0.3, *p* = 0.58; Test and Gender: *F*_(1,26)_ = 0.6, *p* = 0.58, Valence of the Adapter, Test, and Gender: *F*_(1,26)_ = 0.7, *p* = 0.50. In addition, single sample *t*-tests were performed to compare judgments given to the neutral, negative, and positive tests after adaptation to the negative and positive adapters with the mean score to the neutral, negative, and positive tests as obtained in the preliminary rating and none of them were significant (**Figure 3**).

## Discussion

Adaptation and aftereffects have been widely investigated by employing low- (e.g., Suzuki and Cavanagh, 1998) and high-level stimuli (Rhodes et al., 2004). To assess how stimulus properties can be global in an ideal ordering of visual content that produce perceptual aftereffects, in the current study, two experiments were carried out to investigate affective adaptation and valence-specific aftereffects. Specifically, participants were exposed to images with positive and negative affective value and had to rate the valence of subsequent test pictures. Moreover, we used as tests not only images with a neutral affective value, but also images with a positive and a negative value themselves. The presence of faces was intentionally excluded from the sets of pictures employed as adapters and tests, in order to prevent the possibility that any potential effect of affective adaptation could be driven latently by the well-known aftereffects produced by facial emotional expressions. Our results show that participants judged a neutral test picture as more positive after having been exposed to a positive image adapter, while the judgment was shifted to a more negative value after having been previously adapted to a negative image adapter. Moreover, a negative test image was evaluated as more negative following a negative adapter compared to a positive adapter and a positive test image was evaluated as more positive following a positive adapter compared to a negative adapter. It must be acknowledged that these results should be taken with some caution, considering differences in statistical power. The main effect of the test image valence was statistically powerful and that of the adapter image valence was moderately supported. Nevertheless, the interaction between the two factors – central to the current study – was rather weakly supported by statistical power.

These results seem to confirm previous evidence obtained in a variety of methodological studies (priming studies, context processing, rapid serial visual presentation), which covered a number of temporal features of the “adapter” and “test” stimuli (usually short presentations as in priming or rapid serial visual presentation), as well as various levels of stimulus repetition in respect to the nature of the “adapter” (from isolated primes to multiple stimuli streams used as adapters). In the present study, using a typical adaptation procedure, the exposure to a complex picture, with a given valence for 10 s, influenced the subsequent evaluation of the valence of a test picture presented for 500 ms. Consequently, the aftereffect was always assimilative in nature, in that the positive or negative valence of the adapter picture “carried over” the appreciation of the test picture valence. Interestingly, this was manifested in the typical adaptation sequence embedded in our experimental design, where the neutral test pictures were rated as more positive following positive adapters and more negative following negative adapters, in which case it would suggest anassimilative aftereffect of valence. The present results also show that assimilative aftereffects of negative and positive adapter pictures manifested themselves also on non-neutral test pictures. This result might be better framed as a “congruence effect” in the extant literature on priming or affective context processing. Notably, these aftereffects were not driven by the well-known influence of facial expression in emotion adaptation, inasmuch the picture sets were carefully selected to not include faces.

The interpretation that the assimilative aftereffects could be attributed to prolonged stimulus exposure (i.e., adaptation) was supported by the evidence obtained in Experiment 2, wherein the presentation time of the prior stimulus was set to 500 ms. This value is far too short for an adaptation paradigm, and far too long for a priming paradigm. Thus, the null result in Experiment 2 suggests that the adaptation effects observed in Experiment 1 were genuine. Further, it does not seem likely that the long and repetitive nature of the task in the adaptation paradigm had induced effects of learning and fatigue on our participants, because the repetition structure of the non-adaptation experiment was the same as in the adaptation experiment. Adaptation to the emotional content of complex pictures, does not seem to produce contrastive aftereffects as those that have been widely reported for face emotion adaptation (e.g., Rutherford et al., 2008), but rather assimilative “carry-over” or “context” effects as already documented in other methodological studies (Fujimura et al., 2013; Czekóová et al., 2015). It could be speculated that the appraisal of valence, as observed in a complex picture, cannot be considered a “global” feature of automatic perceptual analysis depending on specialized neural units along the visual system. Typically, when such units exist, their (neural) adaptation can explain the phenomenological experience of contrastive aftereffects observed in perceptual adaptation paradigms (Webster and MacLeod, 2011), with stronger aftereffects at earlier levels of visual analysis, corresponding to neural circuitry specialized for hierarchically simpler features. However, contrastive aftereffects have often been observed also for complex visual content or for more global features of certain stimuli (but see Kovács et al., 2006, for a counterexample).A neural locus responsible for the effects of adaptation cannot be assumed as easily as in the case of simpler features, due to the fact that higher-level visual analysis engages multiple and increasingly interconnected regions of the visual brain and cannot depend strictly on the adaptation properties of single neurons. In particular, this is true for the adaptation to the emotional expression of faces, which typically exerts aftereffects that are contrastive in direction, despite the encoding of these stimuli that entails neural networks involving connections extending deeply in the visual brain, to the so-called “affective” and “social” brain (Brancucci et al., 2009). The fact that the typically contrastive aftereffects observed following adaptation to emotional expression of faces are replaced by assimilative effects when the visual content is complex and does not entail the presence of faces as documented in the present study, suggests the idea that prototypical stimulus categories (e.g., faces) do provide a structural framework for parametric encoding along the pathway of perceptual analysis, which is in turn more suitable to support contrastive aftereffects due to neural adaptation of dedicated circuitry (Webster and MacLeod, 2011). In other words, since neural encoding depends on a relatively low number of parameters, it might be hypothesized that for some stimulus categories (like color or facial expression) contrastive aftereffects would reveal the neural adaptation with respect to a predictable distribution of parameters in the space of stimuli (such as the face space) to which the observer is usually exposed. This would not be applicable for the overall perceived valence derived from a complex visual image. In such a case, due to the unpredictable number of objects and their relationships in the picture itself, parameters might be too numerous and unstructured, in which the neural representation might be much sparser than in the case of faces.

Finally, since previous evidence of assimilative context effects using emotional pictures (Fujimura et al., 2013; Czekóová et al., 2015) had not controlled for the relative contribution of the face vs. non-face pictures that were included in the stimulus material, the present results make deeper investigations on the content-specificity of emotional adaptation necessary. This might involve, for instance, comparing the effects of adaptation to two identical sets of visual images differing only for the presence or absence of a person expressing a given emotion.

## Ethics Statement

University of Chieti: The local ethics committee approved this study, and written informed consent was obtained from all participants. All procedures employed in the study comply with ethical standards on human experimentation and with the Helsinki Declaration of 1975, as revised in 2008.

## Author Contributions

RP: experiment design, data collection, data analysis, and manuscript writing. SD: data collection, data analysis, and manuscript writing. AQ: data collection and data analysis. LT: experiment design and manuscript writing.

## Conflict of Interest Statement

The authors declare that the research was conducted in the absence of any commercial or financial relationships that could be construed as a potential conflict of interest.
